# Differential Expression of Antimicrobial Peptides in *Streptococcus pneumoniae* Keratitis and STAT3-Dependent Expression of LL-37 by *Streptococcus pneumoniae* in Human Corneal Epithelial Cells

**DOI:** 10.3390/pathogens8010031

**Published:** 2019-03-06

**Authors:** Prerana Sharma, Natalia Sharma, Priyasha Mishra, Joveeta Joseph, Dilip K. Mishra, Prashant Garg, Sanhita Roy

**Affiliations:** 1 Prof. Brien Holden Eye Research Center, LV Prasad Eye Institute, Hyderabad 500034, India; preranasharma_22@yahoo.co.in (P.S.); natalia_jmf@yahoo.co.in (N.S.); priyashakmishra@gmail.com (P.M.); 2 Department of Animal Sciences, University of Hyderabad, Hyderabad 500046, India; 3 Jhaveri Microbiology Centre, LV Prasad Eye Institute, Hyderabad 500034, India; joveeta@lvpei.org; 4 Pathology Department, LV Prasad Eye Institute, Hyderabad 500034, India; dilipkumarmishra@lvpei.org; 5 Tej Kohli Cornea Institute, Hyderabad 500034, India; prashant@lvpei.org

**Keywords:** antimicrobial peptides, LL-37, STAT3, corneal epithelial cells, *Streptococcus pneumoniae*

## Abstract

*Streptococcus pneumoniae* is the leading cause of bacterial keratitis in the developing world with a growing trend of acquiring resistance against various antibiotics. In the current study, we determined the expression of different antimicrobial peptides (AMPs) in response to *S. pneumoniae* in patients, as well as in primary and immortalized human corneal epithelial cells. We further focused on LL-37 and determined its expression in human cornea infected with *S. pneumoniae* and studied the killing ability of LL-37 against *S. pneumoniae.* The expression of AMPs was determined by quantitative PCR and the phosphorylation of signaling proteins was evaluated by immunoblot analysis. LL-37 expression was also determined by immunofluorescence and Western blot method and the killing ability of LL-37 against *S. pneumoniae* was determined by colony-forming units. Differential expression of antimicrobial peptides was observed in patients with *S. pneumoniae* keratitis. Although *S. pneumoniae* induced expression of the AMPs in human corneal epithelial cells (HCEC), it did not induce AMP expression in U937, a human monocyte cell line. *S. pneumoniae* also caused activation of nuclear factor kappa-light-chain enhancer of activated B cells (NF-κB)and mitogen activated protein kinase (MAPK) pathways in corneal epithelial cells. LL-37 was found to be effective against both laboratory and clinical strains of *S. pneumoniae*. LL-37 induction by *S. pneumoniae* in human corneal epithelial cells was mediated by signal transducer and activator of transcription 3 (STAT3) activation, and inhibition of STAT3 activation significantly reduced LL-37 expression. Our study determines an extensive profile of AMPs expressed in the human cornea during *S. pneumoniae* infection, and suggests the potential of LL-37 to be developed as an alternative therapeutic intervention to fight increasing antibiotic resistance among bacteria.

## 1. Introduction

Bacterial keratitis is a major cause of visual impairment and blindness worldwide [1]. It is characterized by severe pain, inflammation, and corneal opacity. *Streptococcus pneumoniae,* a Gram-positive invasive pathogen, is one of the leading causes of keratitis in India and globally, and it is associated with both trauma and the use of contact lenses [2]. It is also widely responsible for pneumonia, meningitis [3], septicemia [4], and otitis media [5]. Central to the pathogenicity of *S. pneumonia* are pneumolysin [6,7], a pore-forming toxin, autolysin [8], and components of the cell wall [9].

Corneal infections due to *S. pneumoniae* are very aggressive irrespective of antibiotic sensitivity. A recent 10-year trend analysis in antimicrobial resistance among respiratory isolates of *S. pneumoniae* showed an increase in prevalence genotypically [10]. Furthermore, there are recent reports on infections caused by antibiotic resistant strains of *S. pneumonia* [11]. Reports also indicate the growing trend of resistance toward various classes of antibiotics including fluoroquinolones, levofloxacin, and ofloxacin in *S. pneumonia* [12,13]. The increase in antibiotic resistance in ocular isolates of *S. pneumoniae* was also recently reported [14]. This growing prevalence of antibiotic-resistant *S. pneumoniae* is an increasing concern worldwide and, thus, more research for alternative antimicrobial agents is highly required.

The corneal epithelium offers the first line of defense against invading pathogens [15] and mediates innate immune responses by secreting cytokines, chemokines, and antimicrobial peptides (AMPs) through different signaling pathways [16,17]. AMPs are central to the immune responses against pathogens and are mainly secreted by epithelial cells and immune cells. Since AMPs have a broad spectrum of activity and kill pathogens rapidly, it is mostly difficult for the pathogens to develop resistance against them [18,19]. Different classes of AMPs like defensins, cathelicidin, and S100A proteins are known to be expressed in mammals, including humans [20]. Of the several AMPs, we focused on LL-37, the only human cathelicidin, which also has multifunctional abilities including the induction of cytokines, wound healing, and autophagy in cells [21,22]. LL-37, encoded by the *CAMP* gene, is widely expressed by epithelial cells, neutrophils, macrophages, and lymphocytes [23]. It is induced mainly during bacterial infection and inflammation, and it is known to exhibit microbicidal activity, along with ability to influence several innate inflammatory processes and adaptive immune responses [24,25]. *CRAMP*, a mouse homolog to LL-37, deficient mice are reported to be more susceptible to keratitis caused by *Pseudomonas aeruginosa* [26]. Enhanced *Salmonella typhimurium* survival was reported in macrophages obtained from *CRAMP*-deficient mice [27], and these mice were more susceptible to group A *Streptococcus* skin infection [28]. Overall, these accentuate the importance of further studies to understand the role of LL-37 during infections.

In this study, we determined the expression pattern of several AMPs from patients with *S. pneumoniae* corneal infection for the first time, and also in vitro using both primary and immortalized human corneal epithelial cells in response to this pathogen. In addition, we infected U937 cells and checked the expression of AMPs in these cells, since immune cells migrate to the site of infection and secrete AMPs and cytokines. We also found that *S. pneumoniae* causes NF-κB, MAPK, and STAT3 activation in corneal epithelial cells. We further studied the expression of LL-37, the sole member of the human cathelicidin group, by HCEC in response to *S. pneumoniae*, and found that LL-37 expression is mediated by STAT3. We additionally examined the killing effect of LL-37 against laboratory and clinical strains of *S. pneumoniae*. Since antibiotic resistance is on rise, further studies are highly needed to ascertain other therapeutic interventions, and LL-37 might have the potential to be considered as an alternative to combat *S. pneumoniae* infections.

## 2. Materials and Methods

### 2.1. Corneal Scrapings

Corneal scrapings were collected from patients with bacterial keratitis with informed consent. The protocol for obtaining scrapings was approved by the Institutional Review Board of Hyderabad Eye Research Foundation, LV Prasad Eye Institute (LVPEI), India and the research followed the tenets of the Declaration of Helsinki. Cadaveric corneas unsuitable for transplant purposes were used for controls and were obtained from Ramayamma International Eye Bank, LVPEI, India. The corneal tissue sections of *S. pneumoniae* patients were obtained from the Pathology Department, LVPEI.

### 2.2. Identification of Bacterial Strains

Corneal ulcer materials collected aseptically were investigated for bacterial identification at Jhaveri Microbiology Centre, LVPEI, following institute protocol. Briefly, ulcer materials were placed on glass slides for Gram staining and were inoculated in different specific media for bacterial cultures. The pure homogeneous culture was then subjected to Vitek 2 compact (bioMerieux Inc., Durham, NC, USA) analysis for the identification of the bacterium along with Gram staining and a series of biochemical tests.

### 2.3. Bacterial Culture

For bacterial infection experiments, *Streptococcus pneumoniae* American Type Culture Collection (ATCC) 49619 (Sp ATCC) and an ocular clinical isolate (Sp Clinical Strain), obtained from LV Prasad Eye Institute, were used in this study. Both the strains were grown in Todd Hewitt broth (THB) (Sigma-Aldrich, St. Louis, MO, USA) overnight at 37 °C in the presence of 5% CO_2_. Bacteria were grown to mid-exponential phase containing 10^8^ colony-forming units (cfu)/mL, centrifuged at 10,000 rpm for 10 min, and suspended in 1× phosphate buffer saline (PBS) to desired concentration for in vitro experiments. 

### 2.4. Cell Culture

Immortalized human corneal epithelial cells 10.014 pRSV-T [29] were maintained in keratinocyte serum free (KSFM) media containing bovine pituitary extract and recombinant human epidermal growth factors (Invitrogen, Carlsbad, CA, USA) at 37 °C and 5% CO_2_ [30]. HCECs were grown overnight in 12-well plates (1 × 10^5^ cells/well) and incubated with bacteria usually at a multiplicity of infection (MOI) of 1:10 (cells: bacteria) or as mentioned in the text. The cells were then washed with 1× PBS and processed further. Primary human corneal epithelial cells were isolated as described before [31] from donor corneas obtained from Ramayamma International Eye Bank, LVPEI, India. In brief, bulbar conjunctival tissues were removed, and the cornea was incubated in Hanks buffer solution containing dispase and antibiotics at 4 °C. Corneal epithelium was collected by gentle scraping, before being trypsinized, centrifuged, and grown in KSFM containing required growth factors. Cells from passage two were used for experiments. Human monocytes U937 were cultured under similar conditions in RPMI containing 10% fetal bovine serum (FBS), and were infected at an MOI of 1:10, unless mentioned otherwise. Cells were incubated with STAT3 inhibitor, Stattic (Abcam, Cambridge, MA, USA), during some experiments as described in the text.

### 2.5. RNA Isolation, Complementary DNA (cDNA) Synthesis and Quantitative PCR Analysis

The expression of different genes from corneal scrapings, and primary and immortalized cell lines were determined by quantitative PCR. Total RNA was isolated using Trizol (Invitrogen, Carlsbad, CA, USA), and reverse transcription was done using a Verso cDNA Synthesis Kit (Thermo Scientific, Waltham, MA, USA) according to the manufacturer’s protocol. Quantitative PCR was done on an ABI PRISM 7900HT Sequence Detection System (Applied Biosystems, Grand Island, NY, USA) using the SYBR Green PCR Master Mix (Thermo Scientific, MA, USA). The primers used were described in an earlier study [30]. Relative quantities of messenger RNA (mRNA) expression of respective genes were normalized using the 2^−ΔΔct^ method using *GAPDH* as the housekeeping gene.

### 2.6. Western Blot

HCECs were incubated with *S. pneumoniae* for indicated timepoints at an MOI of 1:10 (cells to bacteria). Endogenous phospho-Iκβ, phospho-p38, phospho-ERK, phospho-JNK, phospho-STAT3, and LL-37 protein expression levels were validated by Western blot. The cells were washed with 1× PBS post infection and lysates were prepared using 1× lysis buffer (CST, Danvers, MA, USA); total protein was estimated by the bicinchoninic acid (BCA) method as described in the manufacturer’s protocol (Thermo Scientific, CA, USA). Proteins were separated using 10% or 12% SDS-PAGE, transferred to a nitrocellulose membrane, and probed with p-p38, p-ERK, p-Iκβ, p-JNK, and p-STAT3 (raised in rabbit), and β-actin and LL-37 (raised in mouse) (1:2000 dilution; CST, MA). Subsequently, blots were incubated with IRDye-680 secondary antibody (1:6000 dilution; LI-COR Biotechnology, Lincoln, NE, USA) and were developed on an Odyssey CLx Imaging System (LI-COR Biotechnology, NE).

### 2.7. Immunostaining

HCECs were cultured overnight on coverslips and were infected with *S. pneumoniae* at an MOI of 1:10 (cells to bacteria) for the defined time points, before being processed as mentioned earlier [32]. In brief, the cells were fixed with 4% paraformaldehyde for 15 min, permeabilized with 0.2% Triton X-100, and incubated with anti-LL-37 antibody (1:100; BioLegend, San Diego, CA, USA), followed by incubation with Alexafluor-488-labeled secondary antibody (1:500; Molecular probes). Images were captured on a fluorescent microscope (Olympus IX73, Zeiss, Germany) using the 20× objective.

### 2.8. Immunohistochemistry

Tissue sections (5 μm) of paraffin-embedded corneas diagnosed with *S. pneumoniae* keratitis were obtained along with control cadaveric corneas found unsuitable for transplantation. These sections were deparaffinized and stained with anti LL-37 antibody (1:100; Novus BioLegend, San Diego, CA, USA) as described earlier [33] and were then counterstained with 4’,6-diamidino-2-phenylindole (DAPI) (Abcam, Cambridge) before being observed under fluorescent microscope (Olympus IX73, Zeiss, Germany) using the 20× objective and imaged using Olympus DP71 camera.

### 2.9. In Vitro Susceptibility Test of *S. pneumoniae* with LL-37

Both the ATCC and the clinical strain of *S. pneumoniae* were grown in THB medium, in a CO_2_ incubator at 37 °C until an optical density at 600 nm of 0.28 was obtained. Bacteria were centrifuged at 10,000 rpm for 10 min and were further diluted to 10^4^ cfu/mL and were incubated with increasing concentration of LL-37 (Invivogen, San Diego, CA, USA) in THB medium at 37 °C for 4 h. Serial dilutions of the culture were then plated in blood agar plates and bacterial viability was quantitated by cfu after overnight incubation.

### 2.10. Statistical Analysis

Bar graphs represent means and error bars represent standard error of the mean (SEM). Statistical analysis was performed using either one-way ANOVA or an unpaired *t*-test using Prism7 (GraphPad Software, La Jolla, CA, USA); *p*-values less than 0.05 were considered significant.

## 3. Results

### 3.1. Differential Expressions of Antimicrobial Peptides during *S. pneumoniae* Keratitis

**Figure 1:**
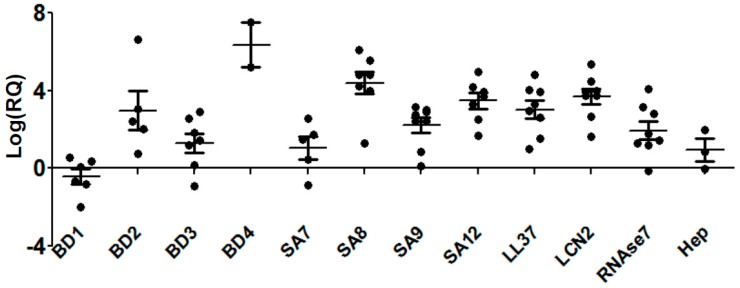
Differential expression of antimicrobial peptides in patients with *Streptococcus pneumoniae* keratitis. Expression of antimicrobial peptides was determined from corneal scrapings of patients infected with *S. pneumoniae* by quantitative PCR, and the values are represented as the log of relative gene expression (log (RQ)) compared to uninfected cadaveric corneas (*p* < 0.0001). Data points represent individual patients.

Expressions of AMPs were reported in several diseases caused by various pathogens like *H. pylori* [34], *P. aeruginosa* [35], mycobacterium [36], and *S. pyogenes* [37]. We also earlier reported the expression of AMPs from patients with corneal infections caused by *P. aeruginosa* [30] and *C. pseudodiphtheriticum* [38]. Here, we report the expression of several AMPs in patients with *S. pneumoniae* corneal infections as determined by quantitative PCR. As shown in Figure 1, AMPs are differentially expressed in patients with *S. pneumoniae* keratitis, presented here as the log of relative gene expression, log (RQ). We obtained corneal scrapings from 12 patients detected with *S. pneumoniae* keratitis, and eight cadaveric corneas were used for controls. The clinical characteristics of the patients are detailed in Table 1. Four members of human beta-defensin (hBD) group were checked, and we observed a significant increase in the expression of *hBD2*, *hBD3*, and *hBD4*. However, the expression of *hBD1* was significantly reduced. The expressions of four AMPs belonging to the group of S100A proteins, *S100A7*, *S100A8*, *S100A9*, and *S100A12*, were also significantly upregulated in these patients, whereby the most increased expression was seen with *S100A8*. There was a significant increase in expression of other AMPs including *LL-37*, hepcidin, lipocalin 2, and Rnase7.

### 3.2. Expression of Antimicrobial Peptides in Immortalized and Primary HCECs and U937 in Response to *S. pneumoniae*

**Figure 2:**
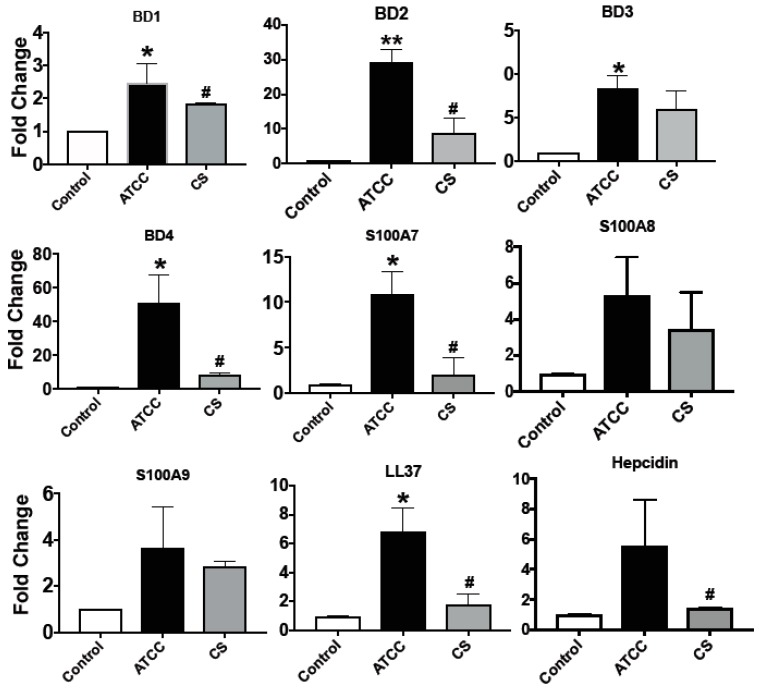
Expression of antimicrobial peptides *in vitro*. HCECs were exposed to *S. pneumoniae* at a multiplicity of infection (MOI) of 10:1 (bacteria:cells), both laboratory and clinical isolates, and expressions of antimicrobial peptides were determined after 4 h by real-time PCR. Uninfected cells were used as a control and *GAPDH* was used as the housekeeping gene for qPCR. The experiment was performed in technical duplicates and was repeated twice. (* *p* < 0.05, ** *p* < 0.005 compared to control; # *p* < 0.05 compared to ATCC).

Although there are few reports regarding activation of Toll-like receptors (TLRs) and inflammasomes, and the production of proinflammatory cytokines by human corneal epithelial cells in response to *S. pneumoniae* [39,40], the expression of AMPs remains to be explored. As we saw differential expression of AMPs in patients with *S. pneumoniae* corneal infections, our aim was to see if *S. pneumonia* infection causes AMP secretion *in vitro*. HCECs were exposed to the bacteria for 3 h followed by 1 h of incubation in gentamycin-containing media to kill any external adhered bacteria; the expressions of AMPs were determined by qPCR. Furthermore, to see if there were any differences in AMP expression in response to clinical strain, we infected HCECs with an ocular clinical strain of *S. pneumoniae* (Sp CS) in addition to the laboratory ATCC strain. Sp CS was positive for pneumolysin, similar to the ATCC strain (data not shown), and was sensitive to all regular antibiotics, except amikacin. As shown in Figure 2, there was significantly increased expression of all the β-defensins studied, particularly that of *hBD2* (30-fold) and *hBD4* (50-fold). The antimicrobial peptides belonging to the S100A group were also expressed by HCECs in response to *S. pneumoniae*. There was more than a 10-fold increase of *S100A7*, and more than a six-fold increase of *S100A8* and *S100A9*. Surprisingly, S100A12, which was significantly increased in patients, was not expressed by HCECs in response to either Sp ATCC or Sp CS. Increased expressions of *LL-37* (six-fold) and hepcidin (five-fold) were also observed in HCECs in response to *S. pneumoniae.* In contrast, the Sp CS infection caused significantly reduced expression of all AMPs compared to the Sp ATCC. The reduced AMP expressions by Sp CS were, however, not due to the induction of cell death, as there was no significant difference in cell viability between cells infected with Sp CS or Sp ATCC (Figure S1A,B).

**Figure 3:**
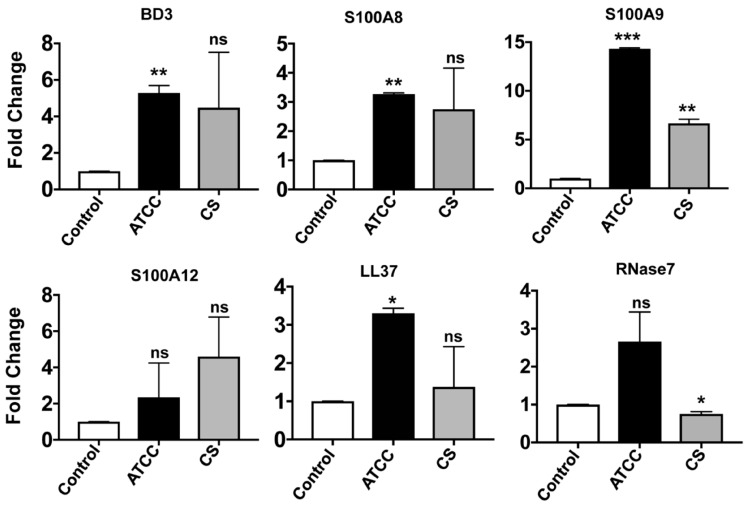
Expression of antimicrobial peptides in primary corneal epithelial cells *in vitro*. Primary human corneal epithelial cells were cultured from donor corneas and exposed to *S. pneumoniae* at an MOI of 10:1 (bacteria:cells), and expressions of antimicrobial peptides were determined after 4 h by real-time PCR. *GAPDH* was used as the housekeeping gene. The experiment was performed in technical duplicates and was repeated three times. (* *p* < 0.05, ** *p* < 0.005, ns is not significant).

We next checked the expression of these AMPs in response to *S. pneumoniae* in primary corneal epithelial cells obtained from donor corneas, as primary cells closely resemble the tissue from which they are derived. Among the β-defensins, expression of only *hBD3* could be detected, which increased significantly in response to Sp ATCC. There were increased expressions of *S100A8* and *S100A9*; however, changes in *S100A12* expression were not significant. Increased LL-37 expression was also observed in primary human corneal epithelial cells in response to Sp ATCC. While there was an insignificant difference in the expression of RNAse7, the expression of hepcidin was not detected in these primary cells in response to *S. pneumoniae*. Except for S100A9, there were no significant changes in the expression of other AMPs by primary corneal epithelial cells in response to clinical isolate Sp CS (Figure 3).

**Figure 4:**
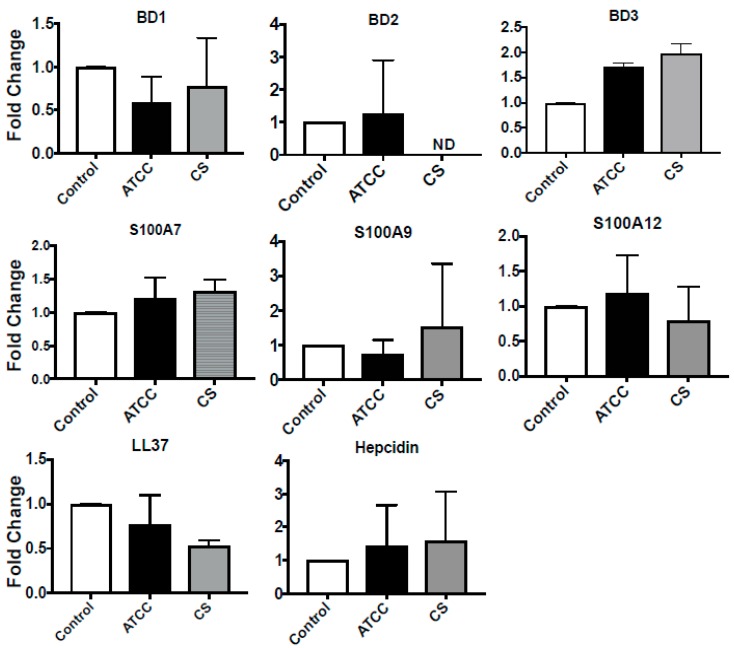
Expression of antimicrobial peptides in U937 *in vitro*. U937 cells were exposed to *S. pneumoniae* at an MOI of 10:1 (bacteria:cells), and expressions of antimicrobial peptides were determined after 4 h by real-time PCR. *GAPDH* was used as the housekeeping gene.

While epithelial cells are the first line of defense, neutrophils and macrophages are prominent immune cells that combat pathogens during ocular infections [41]. Therefore, along with epithelial cells, the expression of AMPs by a human monocyte cell line, U937, was also determined. As shown in Figure 4, there was no significant increase or changes in expression of any of the AMPs by U937 in response to Sp ATCC or Sp CS.

### 3.3. *S. pneumoniae* Induces LL-37 Expression in Human Corneal Epithelial Cells

**Figure 5:**
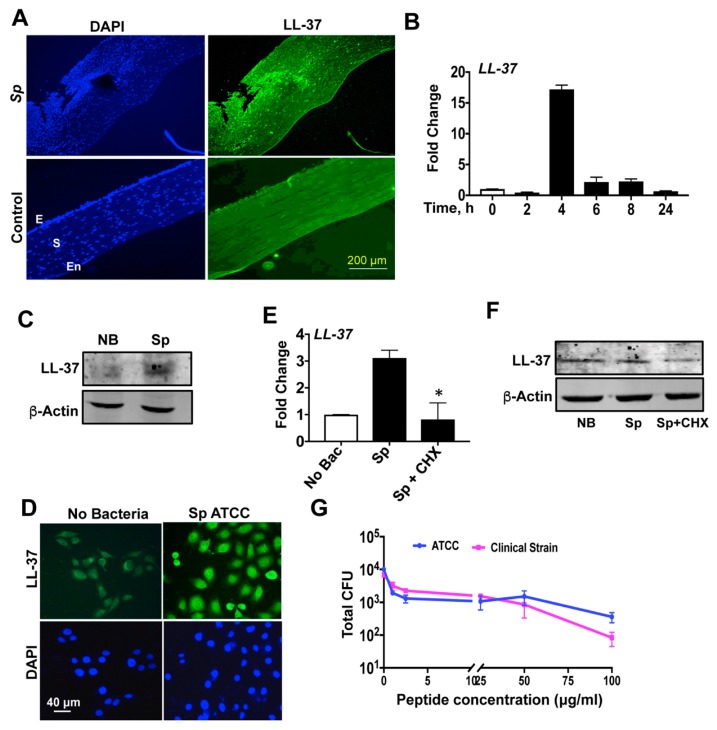
*S. pneumoniae* induces LL-37 expression in human corneal epithelium. Tissue sections from control cadaveric and *S. pneumoniae*-infected corneas were stained with anti-LL-37 antibody followed by Alexafluor 488 secondary antibody and was counterstained with DAPI. The sections were imaged under a fluorescence microscope using the 10× objective. The parts are labeled as follows: E = epithelium, S = stroma, En = endothelium (**A**). Human corneal epithelial cells were exposed to *S. pneumoniae* for defined time periods, and LL-37 expression was determined by quantitative PCR (**B**). LL-37 expression by HCECs in response to *S. pneumoniae* was further determined by Western blot assay using anti-LL-37 antibody (**C**). The expression of LL-37 was also determined by immunostaining. Cells were grown on coverslips and exposed to bacteria at a multiplicity of infection of 10:1 (bacteria:cells) and stained using anti-LL-37 antibody followed by Alexafluor 488 (**D**). *De novo* protein synthesis by HCECs in response to *S. pneumoniae* was inhibited in the presence of cycloheximide (1 μg/mL) and the expression of LL-37 was determined by quantitative PCR (**E**) and Western blot (**F**). Laboratory and clinical strains of *S. pneumoniae* were exposed to different concentrations of LL-37 for 4 h and killing efficiency of LL-37 was determined by the colony-forming unit assay (**G**).

LL-37 is the sole member of the human cathelicidin group; thus, we further investigated LL-37 expression during *S. pneumoniae* infection. We already detected increased LL-37 expression from corneal scrapings of *S. pneumonia* keratitis patients by quantitative PCR (Figure 1). To determine LL-37 expression at the protein level, tissue specimens were obtained from keratitis patients (*n* = 4) who underwent therapeutic penetrating keratoplasty at our institute. As shown in Figure 5A, specific staining for LL-37 was observed in these sections of *S. pneumonia*-infected corneas in both the epithelium and stroma. Cadaveric corneas devoid of any infection were used as the control for the staining experiment. To study the expression of LL-37 in the immortalized corneal epithelial cell line in response to *S. pneumonia,* HCECs were infected with *S. pneumoniae* for different time periods, and the expression of LL-37 was determined by qPCR. The increase in expression of LL-37 was maximum at 4 h post infection, after which there was a decline in the expression, reaching minimum by 24 h post infection (Figure 5B). The expression of LL-37 in response to *S. pneumoniae* was also checked by immunostaining and Western blot analysis, which showed increased expression of LL-37 by *S. pneumoniae* in HCECs (Figure 5C,D). To determine if LL-37 expression by *S. pneumoniae* requires de novo protein synthesis, cells were treated with cycloheximide, a potent translation inhibitor, and LL-37 expression was checked both by qPCR and Western blot. Treatment of HCECs with cycloheximide (CHX) attributed significantly reduced LL-37 gene expression (Figure 5E) and protein levels (Figure 5F) in *S. pneumoniae* infected cells. 

### 3.4. LL-37 Exhibits Antimicrobial Activity against *S. pneumoniae*

Although LL-37 was tested against various clinical isolates [26] of different bacteria, we did not come across any reports involving *S. pneumoniae.* To determine the antimicrobial properties of LL-37 against *S. pneumoniae,* LL-37 was tested against both the ATCC and clinical strains, with concentrations ranging from 0 to 100 μg/mL by colony-forming unit (cfu) assay. Both strains (10^4^ cfu/mL) were incubated with different concentrations of LL-37 for 4 h at 37 °C and were plated in serial dilutions and incubated overnight. LL-37 resulted in a significant decrease in bacterial survival and was effective against both the strains (Figure 5G). 

### 3.5. NF-κB and MAPK Pathways Are Not Responsible for LL-37 Expression by *S. pneumoniaein* HCECs

**Figure 6:**
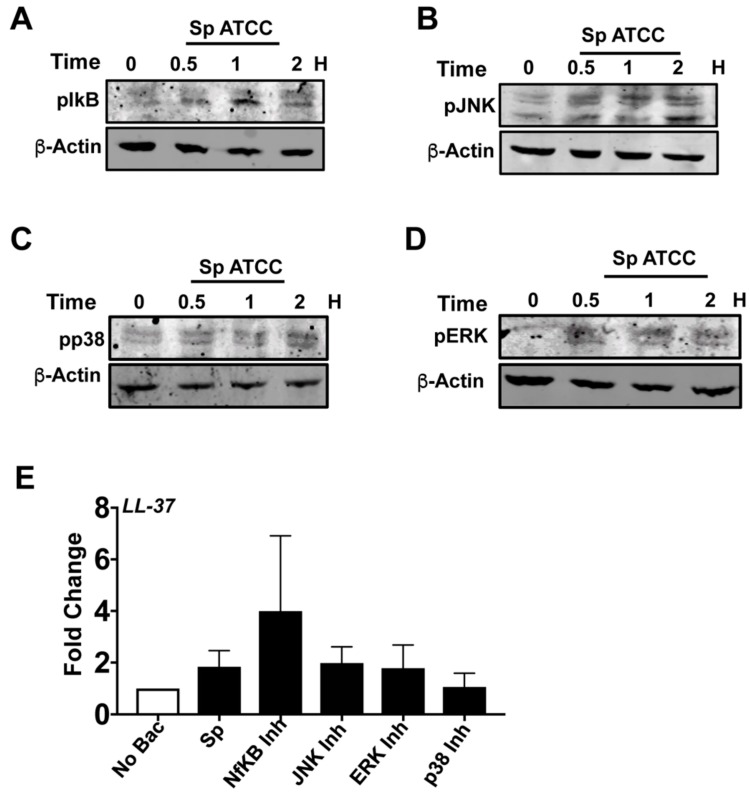
*S. pneumoniae* causes NF-κB and MAPK activation in human corneal epithelial cells. HCECs were exposed to *S. pneumoniae* for 0.5, 1, and 2 h, and expressions of phospho-IκB (**A**), phospho-JNK (**B**), phospho-p38 (**C**), and phospho-ERK (**D**) were determined by immunoblot assay. Immunoblot of β-actin was used as a loading control. HCECs were exposed to *S. pneumoniae* in the presence of NF-κB inhibitor MG132 (10 μM), JNK inhibitor SP600125 (25 μM), p38 inhibitor SB20358 (10 μM), and ERK inhibitor PD98059 (20 μM), and LL-37 expression was determined by qPCR (**E**).

There are several reports regarding the expression of LL-37 mediated by NF-κB and MAPK signaling pathways in different cell lines [42,43]. Therefore, the role of NF-κB and MAPK signaling pathways in *S. pneumonia*-induced LL-37 expression by HCECs was explored using specific inhibitors for NF-κB and MAPK activation. Firstly, we checked if *S. pneumoniae* causes activation of NF-κB and MAPK pathways in human corneal epithelial cells. HCECs were exposed to *S. pneumoniae* for different time points, and the phosphorylation levels of IκB, p38, JNK, and ERK were determined by Western blot analysis. As shown in Figure 6, *S. pneumoniae* induced the phosphorylation of IkB (Figure 6A), JNK (Figure 6B), p38 (Figure 6C), and ERK (Figure 6D) in HCECs by 30 min of infection. Next, to see if any of these signaling proteins mediate LL-37 expression, HCEC were infected with *S. pneumoniae* in the presence of inhibitors for IκB (MG132), p38 (SB203580), JNK (SP600125), and ERK (PD98059). As shown in Figure 6E, there was no reduction in LL-37 expression by *S. pneumoniae* in the presence of these inhibitors, which clearly indicates that expression of LL-37 is not mediated by NF-κB and MAPK pathways in HCEC.

### 3.6. LL-37 Expression by *S. pneumoniaein* HCECs Is Mediated by STAT3

**Figure 7:**
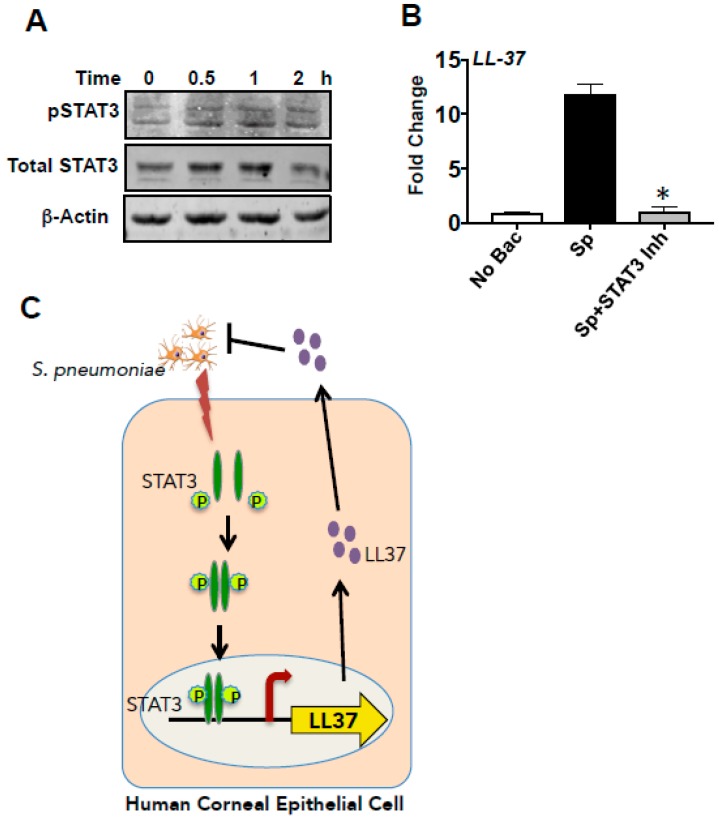
LL-37 expression by *S. pneumoniae* is mediated by STAT3. *S. pneumoniae* causes the activation of STAT3 in a time-dependent manner, which is evident from the phosphorylation of STAT3 in HCECs by Western blot analysis (**A**). HCECs were exposed to *S. pneumoniae* for 4 h in the absence or presence of a STAT3 inhibitor (25 μM), and expression of LL-37 was determined by quantitative PCR (**B**). Representation of proposed mechanism of STAT3 mediated expression of LL-37 by *S. pneumoniae* (**C**).

As shown in Figure 6, *S. pneumonia*-induced LL-37 expression in HCECs was not mediated by NF-κB and MAPK signaling pathways. Further bioinformatics analysis of the promoter region of LL-37 revealed STAT3-binding sites among other transcription factors. Miraglia et al. recently showed that entinostat-induced LL-37 expression was mediated by STAT3 [44]. Likewise, Gombart et al. also previously described different STAT3-binding sites in the LL-37 promoter region [45]. We, therefore, checked if activation of STAT3 might be responsible for LL-37 expression by *S. pneumoniae.* Firstly, we determined the activation of STAT3 by *S. pneumoniae* in corneal epithelial cells. HCECs were exposed to *S. pneumoniae* for 0.5, 1, and 2 h, and phosphorylation of STAT3 was evaluated. Increased phosphorylation of STAT3 was observed within 30 min of exposure to *S. pneumoniae* that continued up to 2 h (Figure 7A). Total STAT3 was also detected, and β-actin was used as a loading control. To confirm that LL-37 induction is mediated by STAT3, HCECs were exposed to *S. pneumoniae* in the presence of a selective inhibitor of STAT3, Stattic. Stattic acts by inhibiting the dimerization and nuclear translocation of STAT3 in cells [46]. We found that the STAT3 inhibitor effectively reduced the expression of LL-37 by *S. pneumoniae* (Figure 7B), indicating that *S. pneumoniae*-induced LL-37 expression is mediated by STAT3. These results, therefore, indicate that *S. pneumoniae* causes phosphorylation of STAT3, which translocates to the nucleus and induces the expression of LL-37 (Figure 7C) in human corneal epithelial cells.

## 4. Discussion

*S. pneumoniae* is the second most leading Gram-positive bacteria causing corneal infections [47] in India. The increase in drug-resistant strains causing corneal infections [48] and the significant economic burden arising from corneal blindness make it essential to identify alternative therapeutic interventions to combat bacterial keratitis. Antimicrobial peptides are one of the most critical candidates that can be developed to overcome antimicrobial resistance among pathogens. Our group earlier reported the expression profile of AMPs in patients with *P. aeruginosa* keratitis [30] and *C. pseudodiphtheriticum* keratitis [38]. In the present study, we have determined the expression profile of endogenous AMPs in corneal ulcers from patients infected with *S. pneumoniae.* Moreover, we showed that *S. pneumoniae* induces LL-37 in a STAT3-dependent manner and that LL-37 is efficient in killing *S. pneumoniae.*

We determined the expression pattern of AMPs elicited in corneal infections caused by *S. pneumoniae* in patients for the first time, and we found significantly increased expressions of hBD2 and 3, S100A8, S100A9, S100A12, LL-37, lipocalin, Rnase7, and hepcidin. Interestingly, the profile elicited by Gram-positive *S. pneumoniae* is markedly distinct from that induced by *P. aeruginosa* [30]. While *P. aeruginosa* caused a significant decrease in Rnase7 expression, there was increased expression of the same by *S. pneumoniae.* The expression of hBD4 was not detected in any patient samples or *in vitro* during *P. aeruginosa* infection; on the contrary, expression of hBD4 was found in both patient samples and in HCECs in response to *S. pneumoniae.* Decreased expression of hBD1 was also seen in *S. pneumoniae* corneal infections compared to elevated expression of the same during *P. aeruginosa* keratitis. These findings are interesting and can probably be exploited in the future to distinguish between Gram-positive and Gram-negative bacterial infection. McIntosh et al. vividly reported the expression spectrum of AMPs by ocular surface cells, such as limbal explant, conjunctival, and corneal epithelial cells [49]. We further checked the expression of the AMPs using primary and immortalized human corneal epithelial cells *in vitro* in response to both laboratory and clinical strains. Increased expressions of AMPs were detected in both primary and immortalized cells in response to *S. pneumoniae.* There are earlier reports of expression of hBD2 by HCECs in response to *S. aureus* in a TLR2-dependent manner [50]. We also previously determined the expression of AMPs in HCECs in response to *P. aeruginosa* [30] and *C. pseudodiphtheriticum* [38], and, while the wild-type PAO1 mostly subverted the expression of AMPs, *C. pseudodiphtheriticum* caused increased expression of S100A peptides and LL-37, but no significant changes in the expression of defensins were detected. However, increased expression of defensins is observed in case of *S. pneumoniae* corneal infections. Corneal limbal epithelial cells were also shown to express several AMPs including hBD3 and RNase 7 in response to *A. castellanii* [51]. Thus, the response of the host cells toward pathogens seems to be very specific for organisms and varies significantly according to the type of insult. In contrast to the increased expression by Sp ATCC, infection by the clinical strain caused reduced expressions of the AMPs in HCECs. A similar observation was determined in primary cells, where increased expressions of AMPs were detected in response to Sp ATCC compared to Sp CS. Several pathogens including *P. aeruginosa* [30], *N. gonorrhoeae* [52], and *S. flexeneri* [53] are known to cause downregulation in the expression of various AMPs. This seems to be the case with the clinical strain of *S. pneumoniae* used in this study. Furthermore, there were no significant changes in the expression of AMPs in response to both the laboratory and the clinical strains in U937. 

Although we found that *S. pneumoniae* induced upregulation of expression of several AMPs, we focused on LL-37 for further studies. LL-37 is known to be expressed by ocular surface epithelia including regenerating corneal epithelium, lung, and gastric epithelial cells [42,54,55] in response to several pathogens. *S. pneumoniae* induced LL-37 expression by lung mast cells [56] similar to what we found with human corneal epithelial cells. Increased LL-37 was also observed in corneal sections obtained from patients with *S. pneumoniae* keratitis in this study. Kinetics of LL-37 expression by HCEC was determined, showing expression peaks around 4 h after which the expression level was lowered. LL-37 was earlier reported to be expressed in response to *M. bovis* in A549 epithelial cells [42] and in soft tissue infections by *S. pyogenes* [37]. Increased LL-37 was also detected and found to be protective in a murine model of *P. aeruginosa* keratitis [57].

The antimicrobial activity of LL-37 was studied in detail against various ocular pathogens like *P. aeruginosa, S. aureus*, and *S. epidermidis* [58]. However, there are no reports of LL-37 activity against ocular strains of *S. pneumoniae*. In this study, the potential bacteria-killing activities of LL-37 were examined against a laboratory and clinical strain of *S. pneumoniae.* Our study indicated that LL-37 is potent against *S. pneumoniae,* both laboratory and clinical strains. LL-37 was also shown to be effective in *S. pneumoniae* meningitis in the presence of ceftaroline [59]. However, in contrast to several reports describing the killing efficiency of LL-37 against various pathogens, Zahner et al. showed that LL-37 can also cause macrolide resistance in *S. pneumoniae* [60].

There are several varying reports regarding the regulation of LL-37 expression, perhaps due to different cell types and in response to varied stimuli. Several studies in the past showed that LL-37 expression is regulated by MAPK signaling pathways. Sayama et al. reported reduced LL-37 expression in the inhibition of the p38 kinase pathway in human keratinocytes. In contrast, Schauber et al. reported the regulation of LL-37 expression by ERK pathways and not by p38 in response to sodium butyrate [61]. Another group demonstrated that LL-37 expression can be regulated by both ERK and p38 activation in response to *M. bovis* in human lung epithelial cells [42]. In the current study, we observed that, although *S. pneumoniae* causes activation of NF-κB and MAPK pathways, LL-37 expression was not mediated by NF-κB, p38, ERK, or JNK. Interestingly, we found that LL-37 expression in HCEC in response to *S. pneumoniae* was mediated by STAT3. This is similar to a recent findings by Miraglia et al., where they showed that entinostat upregulates LL-37 via STAT3 and HIF-1α and not via MAPK pathways in human macrophages [44]. STAT3 was earlier reported to mediate Reg3γ expression in response to methicillin-resistant *S. aureus* [62]. STAT3 is also involved in the expression of hepcidin by interleukin 6 in HepG2 cells [63]. STAT3 was further reported to upregulate β-defensin 2 expression by IL-22 in lung epithelial cells [64].

## 5. Conclusions

In summary, we showed that *S. pneumoniae* elicits a wide array of AMP expression during corneal infections in humans, and induces expression of AMPs in primary and immortalized human corneal epithelial cells. We also observed that LL-37 is effective against *S. pneumoniae,* and that LL-37 expression by *S. pneumoniae* is mediated by the activation of STAT3 and not via MAPK pathways. Taken together, LL-37 might have the potential to be developed as an alternative therapeutic intervention to combat antibiotic resistance and treat bacterial keratitis.

## Figures and Tables

**Table 1:** Clinical characteristics of patients.

Characteristics	*Streptococcus Pneumoniae*
**Age**	25 to 90
Mean (SD)	52.25 (20.176)
**Sex**	
Male (%)	75
Female (%)	25
**Hypopyon**	
Yes (%)	25
No (%)	75
**Occupation**	
Agriculture/Manual Labor (%) Desk Jobs (%)	75 25
**Size of Ulcer**	
<5 mm (%) 5–15 mm (%) >15 mm (%)	37.5 25 37.5

## Supplementary Materials

The following are available online at https://www.mdpi.com/2076-0817/8/1/31/s1: Figure S1: Determination of cell viability of HCEC after *S. pneumoniae* infection.
