# Supplementary material for: Differential Expression of Antimicrobial Peptides in Streptococcus pneumoniae Keratitis and STAT3-Dependent Expression of LL-37 by Streptococcus pneumoniae in Human Corneal Epithelial Cells
Source: Pathogens. 2019 Mar 6;8(1):31. doi: 10.3390/pathogens8010031 (PMC6470555; doi:10.3390/pathogens8010031)
Supplement: Supplementary file 1 [file pathogens-08-00031-s001.pdf]

Figure S1

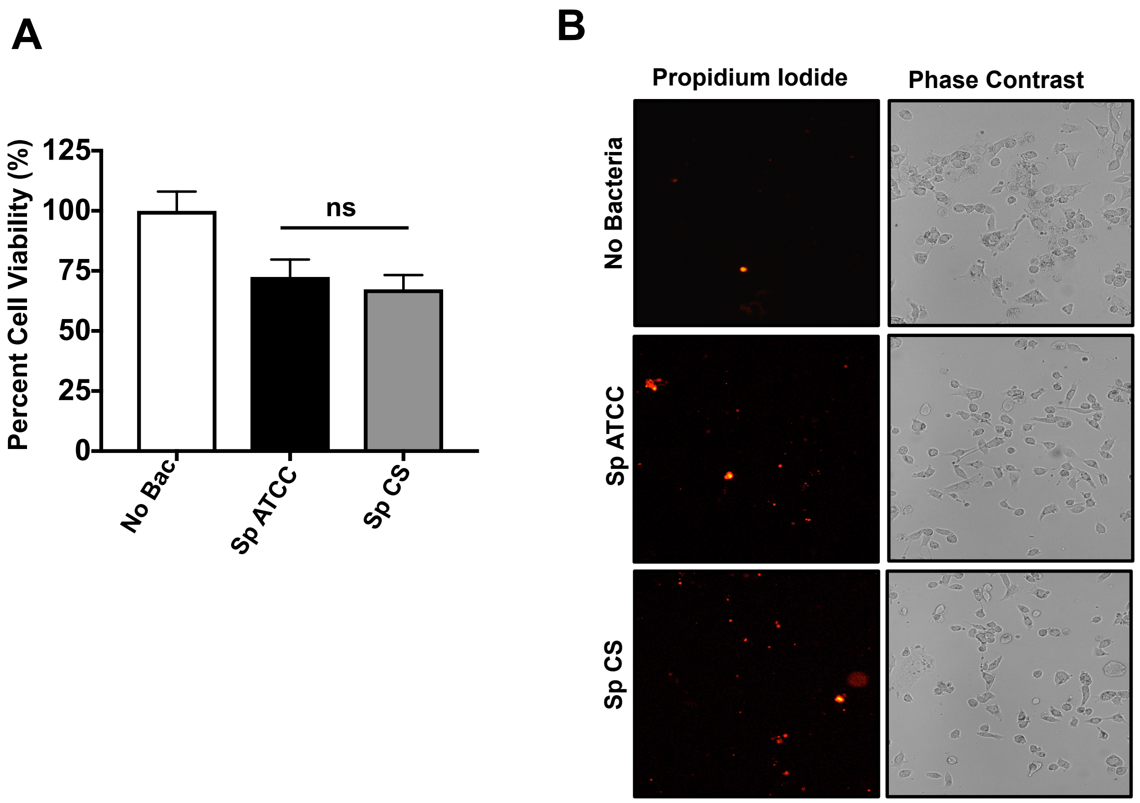

Supplementary Fig. 1: Determination of cell viability of HCEC after *S. pneumoniae* infection. HCEC were exposed to Sp ATCC or Sp CS for 4 h and cell viability was determined by 3-[4,5-Dimethylthiazol-2-yl]-2,5-diphenyltetrazolium bromide (MTT) assay (A) and propidium iodide staining (B). For MTT assay, cells were exposed to bacteria, washed with 1X PBS and incubated with 5 mg/ml MTT for 1 h at 37°C. The supernatant was discarded and formazone crystals were dissolved in DMSO, and absorbance was recorded at 570 nm. For propidium iodide staining, cells were exposed to bacteria, washed and stained with propidium iodide for 15 min, washed and observed directly under microscope using 10X objective. (ns – not significant)
